# Brief History of Syphilis

**Published:** 2014-03-25

**Authors:** M Tampa, I Sarbu, C Matei, V Benea, SR Georgescu

**Affiliations:** * Dermatology Department, “Carol Davila” University of Medicine and Pharmacy, Bucharest; ** Dermatology Department, ”Victor Babes” Hospital for Infectious and Tropical Diseases, Bucharest

**Keywords:** syphilis, syphilis treatment, history of syphilis

## Abstract

Abstract

Before the discovery of Treponema pallidum as the etiologic agent, the origins of syphilis have been the subject of several debates. Diverse therapeutic agents were employed in an attempt to cure the disease. Examining the milestones in the history of syphilis, the present article reviews the existing theories that tried to explain the origins of the disease, the approach in art, the cultural and the evolution of the treatments from the empiric means to the discovery of penicillin.

## Introduction

Syphilis is a sexually transmitted disease caused by Treponema Pallidum, a bacterium classified under Spirochaets phylum, Spirochaetales order, Spirochaetaceae family, but there are at least three more known species causing human treponemal diseases such as Treponema pertenue that causes yaws, Treponema carateum causing pinta and Treponema pallidum endemicum-responsible for bejel or endemic syphilis. The four members of the bacterial family cannot be differentiated with morphological, chemical or immunological methods [**1**,**2**]. Of the aforementioned bacteria, syphilis is the sole sexually transmitted treponemal disease, as the other conditions are transmitted via direct contact with an infected individual [**3**]. 

 From the very beginning, syphilis has been a stigmatized, disgraceful disease; each country whose population was affected by the infection blamed the neighboring (and sometimes enemy) countries for the outbreak. So, the inhabitants of today’s Italy, Germany and United Kingdom named syphilis ‘the French disease’, the French named it ‘the Neapolitan disease’, the Russians assigned the name of ‘Polish disease’, the Polish called it ‘the German disease’, The Danish, the Portuguese and the inhabitants of Northern Africa named it ‘the Spanish/Castilian disease’ and the Turks coined the term ‘Christian disease’. Moreover, in Northern India, the Muslims blamed the Hindu for the outbreak of the affliction. However, the Hindu blamed the Muslims and in the end everyone blamed the Europeans [**4**-**6**].

 In the 16th century, Jean Fernelius, a Parisian teacher whose work and interests were channeled into the mercury treatment of the condition, coined the term ‘lues venera’ (‘venereal pest’) in his treaty dedicated to the affliction [**7**]. Therefore, the term ‘syphilis’ was introduced by Girolamo Fracastoro, a poet and medical personality in Verona. His work “Syphilis sive Morbus Gallicus” (1530) encompasses three books and presents a character named Syphilus, who was a shepherd leading the flocks of King Alcihtous, a character from Greek mythology. In Fracastoro’s tale, Syphilus, mad at Apollo for parching the trees and consuming the springs that fed the shepherd’s flocks, vowed not worship Apollo, but his King. Apollo gets offended and curses people with a hydious disease named syphilis, after the shepherd’s name. The affliction spread to the whole population, including King Alcithous. The nymph Ammerice counseled the inhabitants to offer Apollo further sacrifices, one of which was Syphilus himself, and also to sacrifice to Juno and Tellus, the latter offering the people the tree of Guaiac (Guaiacum officinale), a very used therapeutic medicine in times of Fracastoro [**6**-**8**].

** Hypotheses on the origin of syphilis**

 The pre-columbian hypothesis. The advocates of this hypothesis claim that not only syphilis was widely spread in both Old and New World, but also the other treponemal diseases. In Europe, most of these conditions were mistaken for leprosy [**3**]. According to this hypothesis, pinta occurred in Afro-Asian zone by the year 15.000 BC, having an animal reservoir. Yaws appeared as a consequence of the mutations in pinta around 10.000 BC and spread allover the world, except for the American continent which was isolated. The endemic syphilis emerged from jaws by the selection of several treponemas, as a consequence of climate changes (the appearance of the arid climate) around 7000 BC. Around 3000 BC the sexually transmitted syphilis emerged from endemic syphilis in South-Western Asia, due to lower temperatures of the post-glacial era and spread to Europe and the rest of the world. Initially it manifested as a mild disease, eventually aggravated and grew in virulence, suffering from several mutations, at the end of the 15th century [**2**,**3**].

 The unitarian hypothesis. Considered by some authors as a variant of the pre-Columbian hypothesis, it advocates that the treponemal diseases had always had a global distribution. According to this theory, both syphilis and non-venereal treponemal diseases are variants of the same infections and the clinical differences happen only because of geographic and climate variations and to the degree of cultural development of populations within disparate areas. Briefly, pinta, yaws, endemic syphilis and venereal syphilis are considered as adaptative responses of T. Pallidum to changes in the environment, cultural differences and contact between various populations [**3**,**9**]. In this respect, yaws had a starting point in Central and Western Africa, spreading towards the Iberian Peninsula along with the capturing and selling of Africans as slaves, fifty years before Columbus’ voyage. Yaws, endemic in Africa for that time being, would have remained unmodified in countries with similar climate conditions as those in the origin countries, but would have evolved into endemic syphilis in countries with colder and drier climate in which personal hygiene was overlooked and disregarded and into venereal syphilis in those areas where inhabitants exhibited a civilized society and paid more attention to personal hygiene. The advocates of this hypothesis consider as irrelevant the theory according to which the 44 members of Christopher Columbus crew and the 10 indigenes brought along to Europe could be blamed for syphilis spreading in the whole Europe in just a few years [**10**,**11**].

The Columbian hypothesis. This very popular hypothesis states that the navigators in Columbus fleet would have brought the affliction on their return form the New World in 1493 [**3**,**12**]. This theory is supported by documents belonging to Fernandez de Oviedo and Ruy Diaz de Isla, two physicians with Spanish origins who were present at the moment when Christopher Columbus returned from America. The former, sent by King Ferdinand of Spain in the New World, confirms that the disease he had encountered for the first time in Europe was familiar at that time to the indigenes who had already developed elaborated treatment methods. As for Ruy Diaz de Isla, the physician acknowledges syphilis as an “unknown disease, so far not seen and never described”, that had onset in Barcelona in 1493 and originated in Española Island (Spanish: Isla Española), a part of the Galápagos Islands. Ruy Diaz de Isla is also the one that states in a manuscript that Pinzon de Palos, the pilot of Columbus, and also other members of the crew already suffered from syphilis on their return from the New World [**10**,**12**].

 Ever since, numerous opposites of the Columbian hypothesis tried to prove the pre-existence of syphilis in the Old World, by finding evidence consisting of specific lesions on skeletal remains dated before Columbus journey in America. Radiocarbon dating along with several other modern means of dating, as well as more careful examining of such remains proved that all skeletal parts with specific luetic lesions dated not before, but after 1492. On the other hand, not all skeletal parts evoked by the opposites of Columbian hypothesis were actually exhibiting syphilis lesions. However, in 16 bone fragments the syphilis diagnosis could be certified and modern dating methods showed pre-Columbian origin. Harper et al. explained in an article published in 2011 that all these skeletons were located in coast areas from Europe, in which sea food represented an important part of the inhabitants’ diet. The sea food contained older carbon from the bottom of the ocean that interferes with carbon dating, therefore, after corrections and adequate adjustments have been made, it could be proven that the skeletons actually could not be dated before Columbus return to Europe, as previously considered [**13**].

Unlike Europe, the American continent was able to present clear evidences supporting the existence of syphilis in pre-Columbian period. In this respect, skeletal lesions characteristic for the diagnosis of syphilis which has been identified in various areas plead for syphilis existence in these areas before Columbus discovered America. In addition, radiocarbon dating of the bone fragments showed an age of several thousand years [**4**,**14**].

** Syphilis in Europe**

In 1489, Pope Innocent VIII was in conflict with Ferdinand I of Naples because the Italian King refused to pay his debts to him. As such the Pope offered the kingdom of Naples to Charles VIII who was the Affable of France, and to a certain extent was entitled to reign over this territory by his paternal grandmother, Mary of Anjou. In 1494 Ferdinand I died and his successor, Alfonso II announced his pretences to the Duchy of Milan, which was controlled for a long time by Ludovic Sforza. Ludovic Sforza, in order to avert the threat represented by Alfonso II, encouraged Charles the VIII th to accept the proposal made by Pope Innocent VIII and to conquer the kingdom of Naples. At the end of 1494, one year after the return of Columbus from his first expedition to America, Charles VIII entered Italy with an army of 25.000 men, mainly Flammand, Garcon, Swiss, Spanish and even Italian mercenaries. Initially his army entered Rome, where, for one month, it led a life of limitless depravity. In February 1495 the army of Charles VIIIth entered Naples without encountering any resistance, as the Neapolitan army was made out of no more than 1.000 Italian, German and Spanish mercenaries. The French army was well received by the locals hoping for a better life under French occupation, but later on changed their opinion as they witnessed great flourish in thefts, depravity and mess. The increasing power of Charles VIII leads to an alliance made by the Italian princes, including Ludovic Sforza, who defeats Charles VIII in the battle of Fornovo in July 1495. It is during this battle that the Italian physicians described for the first time a disease they have seen on French soldiers’ bodies, manifested as a generalized eruption consisting of pustules, more terrifying than leprosy and elephantiasis and that could be lethal and was transmitted through sexual intercourse. The disease proved to be syphilis, and the French army was soon blamed for spreading the affliction throughout Italy [**12**,**15**].

 Laura M. Gough, specialist in history of medicine, notes that the war conditions represented a favorable field for the first outbreak of syphilis. It has occurred during Italian invasion by the French armies, in a period of time when all great powers of Europe (France, Spain, the Holy Roman Empire and the Papal States) wanted to gain control over the Apennine Peninsula. As both French and Italian armies were made out of mercenaries brought from the entire Europe, and as the wars lasted for 30 years –a sufficient interval not only for marriages between mercenaries and local females, but also for rape and prostitution-the disease has spread rapidly across Europe as the mercenaries returned to their homeland [**15**,**16**].

 An important aspect to consider is also that syphilis was, at the very beginning, a disease of great severity, a more rapid spreading and atypical in its evolution as compared to nowadays syphilis, the fatal cases were not rare. The supporters of the Columbian hypothesis advocate that the extreme severity if the condition was mainly due to its novelty, as the population had no time to gain any immunity against the ailment as the venereal syphilis became endemic in Europe, certain strains of T. pallidum have selected, and the disease gained a milder course [**17**].

 The spread of the syphilis across Europe was frequently associated with the invasion of Naples by the French army. However, ever since fewer popular theories have been developed. In 1492 Ferdinand de Aragon and Isabel of Castilla issued the Edict of Expulsion of the Jews, stating that all the individuals of Hebrew origins refusing to convert to Catholicism were to be expulsed from Spain and the rest of its territories. On this occasion, approximately 200.000 Jews have left the country for Northern Africa and Southern Europe. On their way, a part of them temporarily settled at the gates of Rome; they were not allowed in Rome, and in the new Diaspora an outbreak occurred, killing 30000 individuals. Despite all efforts, the disease later identified as syphilis entered the city of Rome. Therefore, some of the chroniclers of the time blamed the Jews for the spread of syphilis in Europe; according to them, the disease was already present on Italian territory before Naples invasion by the French in 1495 [**6**,**12**,**17**,**18**].

**Syphilis in artistic representations**

 The oldest artistic representation of syphilis is considered one on a Peruvian jug dating back to VIth century, depicting a mother suffering from syphilis holding a child in her arms; the mother shows a saddle nose and superior incisive teeth with notches on their free margins. The piece belongs to a collection of jugs also encompassing two jugs illustrating leprosy and leishmaniasis [**19**].

**Fig. 1:**
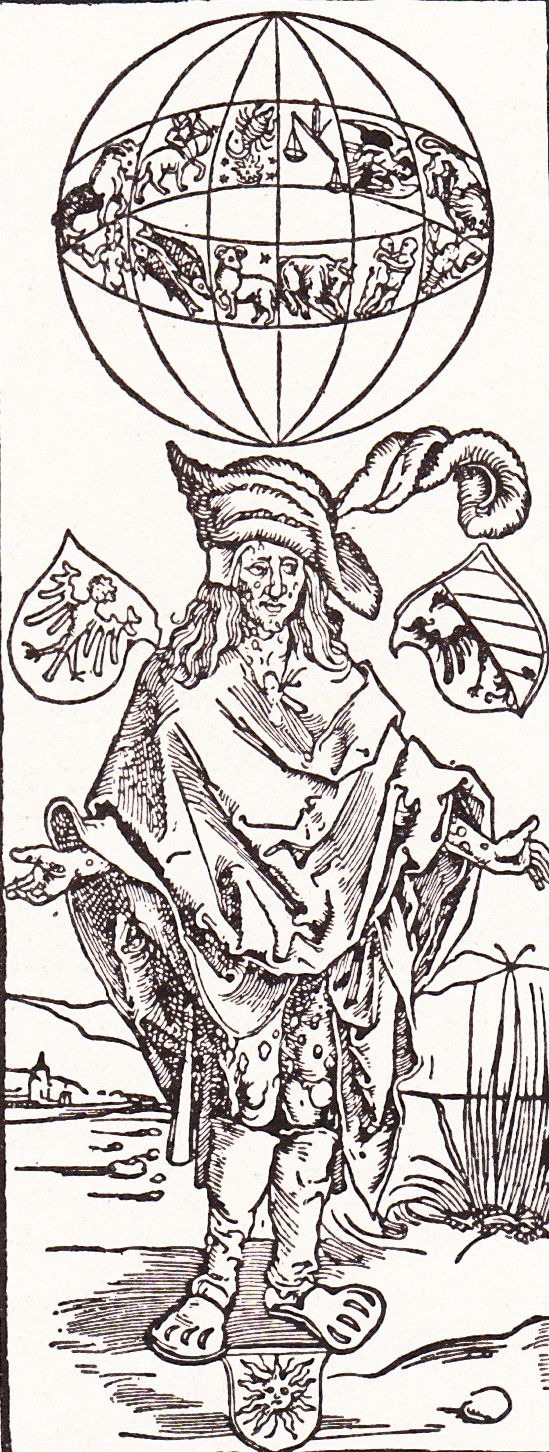
A mercenary whose skin presents multiple chancres, woodcut by Albrecht Dürer, 1496 –published in a Romanian book in 1933 [**21**]

 Albrecht Dürer, a German artist, depicts in woodcuts, for the first time in Europe, in 1496, the image of a mercenary whose skin bears sores of multiple chancres (**Fig. 1**). Next to the image lays written a text by physician Theodorus Ulsenius warning on the new disease, also describing its signs and symptoms, mentioning that the illness is not curable and establishing a direct link between the epidemic and the grand astrological conjuction in 1484 [**19**,**20**].

**Fig. 2:**
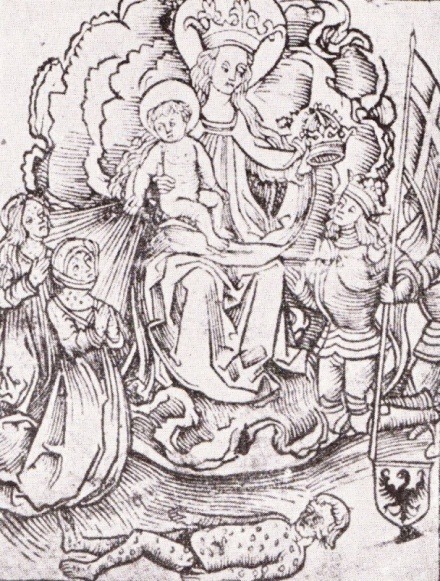
Saint Mary and the Holy Child punishing the sufferers of syphilis, woodcut, 1496 [**21**]

A work of Sebastian Brandt depicting Saint Mary with the Child dates from the same year, 1496 (**Fig. 2**); the Holy Child throws light spears to punish or cure the sufferers of syphilis, represented as individuals with multiples ulcers and sores. In another part of the woodcut depiction, King Maximilian I and his Knights are ready to receive the crown as reward for the edict he issued in 1495, stating that the disease was a consequence of blasphemy and sins, punishing the suffers of syphilis for their immoral behavior [**15**,**19**,**22**].

**Fig. 3:**
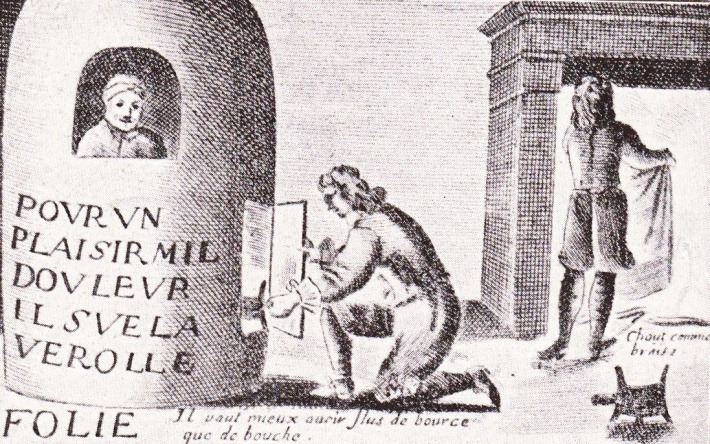
Depiction of a man in a fumigation stove, engraving by Jacques Laniet, Paris, 1659, as published in a Romanian book in 1933 [**21**]

Another significant painting belongs to Jacques Laniet, in the XVIIth century, an illustration of a man in a fumigation stove, a fashionable method of treatment at that time (**Fig. 3**). On the barrel lays written “pur un plaisir, mil douleur” (fr. correspondent of the phrase “For a pleasure, a thousand pains”), a proverb very used in those years. Another verbatim translation of this proverb is “for a night with Venus, a life with Mercury” [**23**].

“The allegory of syphilis”, a work of Luca Giordano depicting Venus and a young man with alopecia and crusted papules on the scalp, as well as collapsed nose bone, and “Warning against syphilis” painted by Johan Sadeler presenting Venus giving birth to a contaminated milk spring from her breasts and a shepherd -allusion to Francasto’s poem on the origins of syphilis [**23**].

 The autoportraits of Gerard de Lairesse (1641-1711), as well as his portrait painted by Rembrandt in the XVIIth century highlight in a clear manner the congenital syphilis sequelae of the Dutch painter, amongst which, frontal bossing and saddle nose [**24**].

 Eduard Munch (1863-1944), an expressionist Norwegian painter presents in “The inheritance” the image of a young mother in tears holding a child with syphilis stigmata in her arms. The painting was initially named “the syphilitic child. According to some critics, Munch was ridiculing in his painting the iconic image of Madonna and the child. Pablo Picasso (1881-1973) was also impressed by this disease. His work “The Wages of Sin” is an allegory of venereal diseases and presents a sailor among prostitutes and a medical student holding a skull, as the symbol of death. This work represents actually a sketch of Les Demoiselles d’Avignon (1907), in which the artist gives up the male characters of the painting and illustrates solely the women who are no longer feminine and beautiful, but unattractive. Some critics believe that this painting points out the artist’s fear of venereal disease [**25**].

** Famous historical figures diagnosed with or strongly suspected of syphilis**

The transmittance of sexual diseases has always been associated with promiscuity and vulnerable population groups [**14**]. However, the rapid spreading proved not to avoid any geographical, racial or social barriers, affecting also great personalities along the history.

The writers were among the most affected category which was more likely, due to the promiscuous and bohemian life, to have the disease. Alphonse Daudet, Thomas Chatterton, Keats, James Boswell, Baudelaire, Heinrich Heine, Dostoievski and Oscar Wild are only a few examples of writers suffering from syphilis. Romanian poet Mihai Eminescu was diagnosed with syphilis too. He died in a mental institution at the age of 39 years [**26**-**28**]. Even the philosophers, who were usually considered superior minds, insensitive to women charms, also have suffered for syphilis. The most famous of them were Friedrich Nietzsche (1844-1900) and Arthur Schopehnauer (1788-1860). The last has contracted the disease while still a student, and in time, developed a tendency to misogyny [**26**].

Famous Casanova is widely and iconically known for his excellent techniques of seduction. However, most of his sexual partners were actually prostitutes, and this may be the reason why he had gonorrhea four times, cancroids for five times, syphilis and genital herpes [**26**,**28**].

It is also presumed that famous painters as Eduard Manet, Paul Gauguin, Vincent van Gogh and Goya, as well as composers like Ludwig van Beethoven, Robert Schumann and Franz Schubert suffered for syphilis [**26**].

Furthermore, the monarchy was not spared of the implacable disease. One example is Tsar Ivan IV Vasilievici (known in history as Ivan the Terrible), Prince of Moscow (1530-1584) who contacted syphilis after the death of his wife. Some authors blame syphilis for his brutal behavior [**26**,**28**,**29**]. King Henry III and Charles V of France, Henry VIII and George IV of England, Paul I of Russia and Maximilian I of Holy Roman Empire are other examples.

Al Capone, the famous gangster who led a crime syndicate in the times of Prohibition in the United States supposedly died of neurosyphilis, as a consequence of aggravation of its manifestation after his imprisonment in Alcatraz [**28**,**29**].

** The history behind the establishment of the etiology of syphilis**

From the very beginning numerous theories on the origin of syphilis existed, most of which linking initially syphilis and leprosy together. According to several fables of the early XVI th century, syphilis was the result of a sexual relation between a Spanish prostitute and a leper. The prostitute also infected the soldiers of Charles VIII. Paracelsus (1493-1541) considered that syphilis was the result of a sexual intercourse between a prostitute suffering from gonorrhea and a French leper. In compliance to other theories of the time, the disease might have been the outcome of the relationship of a prostitute having a uterine abscess with a leper or the result of poisoning the wine with blood coming from a leper [**6**].

Sexual transmitted diseases were seen as a single disease for many centuries. The differentiation between gonorrhea, cancroids and syphilis as distinct maladies was achieved no earlier than XIXth century. In the beginning of XVIIIth century there were several doctors who treated syphilis and gonorrhea as separate entities. However, in 1767 John Hunter a famous physician of venereal diseases at that time (1728-1793) conducted an experiment consisting of an inoculation of the urethral secretion of a gonorrhea patient in the prepuce of a healthy patient, the last developing syphilis shortly afterwards. Consequently, his experiment proved that syphilis resulted from gonorrhea. What Hunter has missed out was that the patient from whom the urethral secretion was taken had both syphilis and gonorrhea. However his experiment, widely acknowledged in his époque, delayed the differential diagnosis of the two diseases with a few decades [**7**,**30**].

 In 1831 Ricord has designed a larger study on syphilis and gonorrhoea and succeeded to show that the last occurs only after contact with gonorrohea patients, whilst the former –only after contact with syphilis patients [**7**].

It was not earlier than 1905 that Schaudinn (1871-1906) and Hoffman (1868 – 1959) have discovered the etiologic agent of syphilis, whom they have named Spirochaeta pallida, on various syhilis lesions, proving its existence in both fresh and Giemsa coloured specimens. It was them who changed the name of the bacterium subsequently to Treponema pallidum [**7**,**29**,**31**].

 In 1906 Landsteiner introduced the use of the dark-field microscopy method for the detection of the spirochete of syphilis. In 1910 the German bacteriologist August Wasserman (1866-1925) came with the first serologic test for syphilis and in 1949 Nelson and Mayer have conceived Treponema pallidum immobilization test (TPI), the first specific test for T. pallidum [**29**,**32**]. Their discoveries had a very important role in detecting the disease in patients who were suspected of syphilis, as well as in other healthy individuals, and in monitoring syphilis response to treatment.

**Syphilis treatments along the history**

**Fig. 4:**
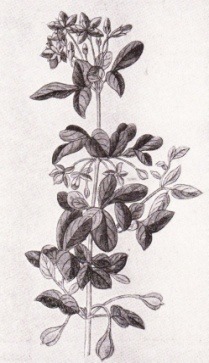
Guaiacum officinale (guaiac tree), used widely for the treatment of syphilis [**21**]

Initially, the treatment of syphilis included less efficient methods that were accompanied by pain and multiple adverse reactions [**29**]. Taking into consideration that the disease was associated with the discovery of the American continent, numerous treatments included plants brought from the New World, such as the guaiac tree (lat. Guaiacum Officinale), known also as sasafras or willow (Salix), which led to the widest recognition at the time (**Fig. 4**). These plants acted as purgative agents, lead to sudoration, diarrhea and the increase in urinary debt and were believed to be “blood cleansers” [**7**]. One of the main supporters of the guaiac tree utilisation in the treatment of syphilis, and in the same time an ardupus opponent of mercury treatment, was Ulrich von Hutten (1488-1523), a former priest who described in a detail the manifestations of the disease as well the simptoms of mercury intoxication, based on his own experience as a sufferrer from the disease [**15**,**33**]. From the guaiac tree a decoction was made, the resulted potion was boiled and the patient was assumed to consume the mixture daily for 30 days. Before drinking the potion, the patient was covered in blankets in order to induce perspiration, and a mild purgative was also administered [**15**].

Paracelus (1493-1541) was one of the first supporters of the mercury treatment. Mercury was used in Arabic medicine in the treatment of several dermatomes as well as leprosy, and succeeded to gain rapidly an important place in medical field at that time. Mercury is a potent diuretic that also leads to excessive salivation when administered in toxic doses. At the time, is was considered that the “virus” was eliminated from the body through sweat, salivation and diuresis [**15**,**33**,**34**]. There were several methods of administering mercury. Thus, in a mixture with grease, mercury was administered topically, leading to ulcerations. Barbarossa pill, named after the Turk admiral who gave the pill to his soldiers and was affected by the malady, contained a mixture of mercury and perfume essence and fruit flavours [**7**]. Mercurous chloride (calomel, Hg2Cl2) was a white salt able to be administered orally, topically or by injections. The metallic form of mercury was administered also under the form of therapeutic fumigation. Patients seemed to find the adverse reactions of the treatment acceptable, however, the treatment lead to systemic intoxication (hidrargirism) and pneumonia [**33**].

Bismuth salts were introduced in syphilis treatments in 1884. These compounds were less toxic than mercury, in the same time bearing a stronger bactericidal effect that the former, consequently, becoming the cardinal heavy metal employed in syphilis treatment [**33**].

**Fig. 5:**
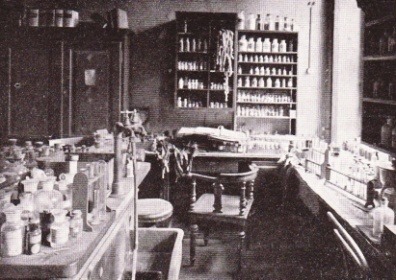
The laboratory in which famous 606 compound was invented [**21**]

German scientist Paul Ehrlich (1854-1915) received Nobel Prize in Physiology and Medicine in 1908 for his discovery of arsphenamine (Salvarsan). The scientist discovered the compound that acted like an antibiotic by accident, while working on finding a cure for Trypanosoma brucei. Ehrlich’s desire was to discover a “magical bullet”- a drug able to specifically bind to a bacterium and kill it, without affecting human cells. Salvarsan was also denominated as “Compound 606”, as it was discovered after 606 failed experiments (**Fig. 5**) [**7**,**35**,**36**]. The safer novel drug that superseded the more toxic and less water-soluble salvarsan as a treatment for syphilis was Neosalvarsan, also an arsenic compound. Both Salvarsand and Neosalvarsan were replaced in the treatment of syphilis by Penicillin, after 1940.

Observing that fever lead to symptomatic improvement of neurosyphilis, various methods of fever induction have been experimented with turpentine, tuberculine, mercury and even Salmonella typhi. In 1917, the Austrian physician Julis Wagner-Jauregg (1857-1940) includes malaria in the treatment of syphilis. Malaria induced fever paroxysms able to be controlled, as quinine had already been discovered. Jauregg injected patients suffering from malaria with blood presenting Plasmodium vivax. The patients exhibited fever paroxysms lasting for approximately 6 hours and core temperature returning to normal values; quinine was injected after 3-4 cycles on a 2 day period, in order to treat malaria. In 1927 Jauregg received Nobel Prize for Physiology and Medicine for his discovery [**29**,**37**].

In 1928, Alexander Fleming (1881-1955) discovered penicilin and from 1943, it became the main treatment of syphilis [**7**,**29**]. Nowadays, worldwide, prevention and treatment programs control syphilis spreading.

## Conclusions

Treponema pallidum infection in men not only provoked a disease that represented a threat to humans for many centuries, but it also had a tumultuous history. From examining it, one could learn how easy was to place stigma not only on individuals affected by the disease, but on entire nations, as countries were blamed along the history for the spread of the disease. Throughout the centuries, syphilis has affected individuals of various origins, from monarchs, painters and philosophers to low income people, mainly due to promiscuity. Various treatments to cure the disease were tried along the centuries; nowadays, penicillin and prevention programs control the disease. 
